# Preclinical Evidence for the Role of the Yin/Yang Angiotensin System Components in Autism Spectrum Disorder: A Therapeutic Target of Astaxanthin

**DOI:** 10.3390/biomedicines11123156

**Published:** 2023-11-27

**Authors:** Ayat I. Samra, Ahmed S. Kamel, Dalaal M. Abdallah, Mai A. Abd El Fattah, Kawkab A. Ahmed, Hanan S. El-Abhar

**Affiliations:** 1 Pharmacology and Toxicology Department, Faculty of Pharmacy, Cairo University, Cairo 11562, Egypt; dr.ayat.samra@gmail.com (A.I.S.); dalaal.abdallah@pharma.cu.edu.eg (D.M.A.); may.galal@pharma.cu.edu.eg (M.A.A.E.F.); 2 Pathology Department, Faculty of Veterinary Medicine, Cairo University, Cairo 11562, Egypt; kawkababdelaziz@yahoo.com; 3 Pharmacology, Toxicology, and Biochemistry Department, Faculty of Pharmacy, Future University in Egypt (FUE), Cairo 11835, Egypt; hanan.elabhar@fue.edu.eg

**Keywords:** astaxanthin, Notch1 receptor, renin–angiotensin system, autism spectrum disorder, gliosis, tauopathies, Mas receptor, carotenoids, valproic acid, rats

## Abstract

Autism spectrum disorder (ASD) prevalence is emerging with an unclear etiology, hindering effective therapeutic interventions. Recent studies suggest potential renin–angiotensin system (RAS) alterations in different neurological pathologies. However, its implications in ASD are unexplored. This research fulfills the critical gap by investigating dual arms of RAS and their interplay with Notch signaling in ASD, using a valproic acid (VPA) model and assessing astaxanthin’s (AST) modulatory impacts. Experimentally, male pups from pregnant rats receiving either saline or VPA on gestation day 12.5 were divided into control and VPA groups, with subsequent AST treatment in a subset (postnatal days 34–58). Behavioral analyses, histopathological investigations, and electron microscopy provided insights into the neurobehavioral and structural changes induced by AST. Molecular investigations of male pups’ cortices revealed that AST outweighs the protective RAS elements with the inhibition of the detrimental arm. This established the neuroprotective and anti-inflammatory axes of RAS (ACE2/Ang1-7/MasR) in the ASD context. The results showed that AST’s normalization of RAS components and Notch signaling underscore a novel therapeutic avenue in ASD, impacting neuronal integrity and behavioral outcomes. These findings affirm the integral role of RAS in ASD and highlight AST’s potential as a promising treatment intervention, inviting further neurological research implications.

## 1. Introduction

Autism, a neurodevelopmental disorder, is classified as a “spectrum” disorder (ASD) since it displays a range of symptoms during early childhood [1], including motor challenges [2], abnormal social communication, and restricted/repetitive movements [3,4] due to alterations in different brain areas [2]. Indeed, cortical/subcortical overgrowth and dysfunction were detected in post-mortem samples [5].

ASD originates from genetic, environmental, and mother-and-child-associated risk factors [4,6]. The prenatal environment includes exposure to harmful substances, infections, and medication use [7], such as in the case of valproic acid (VPA), which is used in neurological and psychiatric disorders. VPA in pregnancy can cause ASD [8], initially known as fetal valproate syndrome [9,10], partly by increasing oxidative stress, which is detected in ASD patients [11,12].

ASD is not a standalone disorder but is associated with other neurological conditions such as anxiety disorders [13], depression [14], and epilepsy [15,16]. In addition, ASD is highly engaged with Alzheimer’s disease (AD), sharing common characteristics despite impacting individuals at different stages of life [6]. Similar symptoms as dementia, cognitive impairment, speech difficulties, and intellectual disability are common in both diseases, in addition to the presence of different signaling pathways such as the Notch signal [17] and the processing mechanism of amyloid-β precursor protein (APP) [18], highlighting their overlapping pathophysiology. Recently, the renin–angiotensin system (RAS) has participated in the pathophysiology of AD and other neurodegenerative diseases [19], but not in ASD so far. Moreover, the aberrant phosphoinositide 3-kinase (PI3K) signaling pathway contributes to ASD pathogenesis since the PI3K/protein kinase B (Akt) axis has a crucial role in neuronal morphogenesis and survival [20,21]. The cardiogenic effect of the Ang 1-7/Mas receptor was linked to the PI3K/Akt survival signal; however, Mas receptor, PI3K, or Akt inhibitors unequivocally abolish the beneficial effects [22].

Furthermore, accumulated amyloid-β (Aβ) plaques and the presence of hyperphosphorylated tau (p-tau) protein characterize AD [23], as well as autism [24,25]. Therefore, it is noteworthy to understand the tau pathomechanisms in autism and to delve deeper into the effects of reducing tau levels on neurogenesis since its reduction prevents excessive brain growth (megalencephaly) and alleviates behavioral abnormalities, which are facts that nominate it as a targeted therapy against ASD [26].

To date, p-tau is known to be augmented by different kinases, including activated glycogen synthase kinase (GSK)-3β and cyclin-dependent kinase 5 (CDK5) [27]. GSK-3β is activated when the PI3K/Akt axis is turned off [21,28], whereas the effect of CDK5 is mediated by calpain-dependent cleavage of p35 to give the active p25 moiety [21,25]. However, CDK5 enhances learning and memory by playing a role in synaptic plasticity when bound to p35 [29]. Apart from these kinases, our comprehension of the mechanisms underlying tau hyperactivation remains incomplete. 

For example, Notch signaling is implicated in AD and ASD [17]. Moreover, neurofibrillary tangles in AD brains contain Notch1, which overlaps with p-tau in plaque-like structures [30]. A previous study suggested a potential association between tau aggregates and Notch-1 expression in neurodegenerative diseases [31]. 

Furthermore, limited research has been conducted on the involvement of the renin-angiotensin system (RAS) in this process. In one study, the activation of the angiotensin II (Ang II)-converting enzyme (ACE) enhanced p-tau, while its inhibition decreased p-tau [32]. A subsequent study clarified that Ang II activates tau by stimulating CDK5, which is reversed by losartan [33]. Nonetheless, the activated Yang arm of RAS (ACE2/Ang1-7/Mas receptor hub) reduced tau pathology in both AD and Parkinson’s disease (PD) models [34,35]. In addition, AD, with its hallmark p-tau, is linked to decreased ACE2 [36]. Although not extensively studied in autism, one study linked ASD to ACE genetic variations [37]. 

Although the search for a specific pathology underlying ASD is still challenging, recent research suggests that glial cell pathology may be a defining feature of ASD. Astrocytes, the most abundant glial cells in the brain, play a crucial role in neuronal function during development and adulthood [12]. An altered astrocyte number and function have been linked to impaired connectivity, highlighting the potential role of activated astrocytes in neuronal disorders, including ASD [38,39]. Though limited, the available data indicates that while the number of astrocytes is reduced in ASD, their activation level and hallmark glial fibrillary acidic protein (GFAP) expression are increased [12]. Similarly, GFAP is elevated in the cerebrospinal fluid of ASD patients [40]. However, further research is necessary to better understand the role of astrocytes in ASD. Autistic patients with increased levels of secreted APP-α show increased GFAP expression and Notch1 alteration [41]. Furthermore, the activated Notch1/Hes axis promotes the phosphorylation of signal transducer and activator of transcription 3 (STAT3), which is responsible for the expression of GFAP [42,43]. While no correlation was found between RAS and GFAP in neuronal disorders, a previous study reported that the renin inhibitor (aliskiren) reduced LPS-induced astrogliosis in a depression model [44].

Although recent studies highlighted crosstalk between RAS and Notch1, few data are available on their link to neuronal disorders, autism, tauopathy, or astrogliosis. In an in vitro study, Ang II upregulated Notch1 to mediate its harmful effect on renal podocytes [45], while the activation of angiotensin receptor 1 (AT1R) activated the Notch pathway in human vascular smooth muscle cells [46].

ASD lacks reliable treatments due to its diverse pathomechanisms [47]. Hence, preclinical studies are necessary to find new therapies and evaluate their potential mechanisms. Astaxanthin (AST), a natural xanthophyll carotenoid, may hold promise as an adjunctive treatment for autism and neurodegenerative diseases. AST has potent antioxidant capabilities [48] and can cross the blood–brain barrier [49], characteristics that make it a potential option, as proven in a VPA-induced ASD model [50]. However, more data are needed to unveil additional mechanisms beyond its antioxidant role in autism. 

We investigated the potential of AST in treating tauopathy and astrogliosis associated with ASD. We hypothesize that AST’s modulation of the Yin/Yang arms of RAS and correction of the inflammatory signal may prove effective. We used a prenatal VPA-induced ASD model in rats to explore this hypothesis. Indeed, animal models are essential in understanding ASD since they mimic certain traits observed in ASD patients. 

## 2. Materials and Methods

Animal models play a pivotal role in elucidating the intricacies of ASD. These models are reliable in translational research to replicate clinical behavioral and neurobiological traits of ASD. Moreover, as highlighted previously, they are instrumental in exploring potential therapeutic avenues such as mTOR, GABAergic, and glutamatergic systems [51,52]. These insights have spurred efficient interventions in reversing behavioral anomalies in these models. A comparative analysis drew intriguing parallels between clinical observations in ASD patients and findings in animal models [52]. In that review, various ASD animal models, including environmental-induced (e.g., VPA), immune-induced (e.g., maternal immune activation), and genetic (e.g., Shank3 deletion) models, were examined. Among these, the VPA model produced the most pronounced and severe phenotypes across behavioral and cellular aspects with gender relevance, especially to males. This model effectively simulated the consequences of prenatal exposure to certain environmental factors, such as medications, on neurodevelopmental outcomes. However, the review acknowledged the limitations of animal models, emphasizing that not all ASD symptoms, such as cognitive stereotypy, intellectual disability, and speech deficits, are reproducible in rodents.

Moreover, animal models prove to be a critical aspect in understanding the comorbidities often accompanying autism. These comorbidities, like epilepsy [53], mood disturbance [54], and other psychiatric disorders [55], have been noted in animals and have been attributed to the lens of disturbances in the tryptophan–kynurenine metabolic pathway [55].

In our quest to adopt a reliable, reproducible, ethically sound, and methodologically valuable approach to investigating the complex mechanisms underlying ASD, we utilized the in utero VPA model. Specifically, the use of VPA during pregnancy, a prevalent strategy for managing central disorders, often leads to fetal valproate syndrome, reinforcing the ecological validity of this model’s findings. In this study, a methodological decision was made to utilize male offspring to parallel the gender ratio discrepancy observed in ASD prevalence, where males are significantly more affected than females. This is compatible with [55], who documented the behavioral traits of ASD in male offspring more than in female offspring. 

### 2.1. Animals and Induction of the ASD Model

After one week of acclimatization at the institutional animal facility (Faculty of Pharmacy, Cairo University), adult 8-week-old male and female Wistar rats (200–250 g; El-Salam Breeding Center, Cairo, Egypt) were allowed to mate. Nine females were examined every morning for the presence of vaginal plugs or spermatozoa in vaginal smears to confirm pregnancy. Once confirmed, the date was designated as gestational day 0.5 (G 0.5) [56]. A well-established, reliable, and integral autism model, namely, the in utero-VPA paradigm, was used to induce neurotypical autistic signs in offspring, following previous studies [57,58,59,60], in which 6 pregnant female rats received a single intraperitoneal injection of 500 mg/kg sodium VPA (Depakine; SANOFI, Paris PA, France) [57,58,59,60] at the critical period of exposure to VPA [61]. Similarly, the other 3 normal control pregnant female rats received an equal volume of distilled water. The experimental protocol was followed with minor modifications regarding the housing of the animals. Instead of single caging, dams were maintained with their partner till the weaning of the offspring to avoid cannibalism or maternal death noted in our pilot study, resulting from pre-gestational stress, which increases vulnerability to depression [62]. Pups were weaned on postnatal day (PND) 23, and the male offspring enrolled in our study were separated into new cages until the end of the experimentation. Notably, all rats were kept under controlled environmental conditions of constant temperature (23 ± 2 °C), humidity (60 ± 10%), and light/dark cycle (12/12 h; lights on at 6:00 a.m.) and were allowed free access to standard chow pellets and water ad libitum. The handling of animals strictly adhered to the Guide for the Care and Use of Laboratory Animals protocol (NIH publication No.85-23, 2011) adopted by the Research Ethics Committee of the Faculty of Pharmacy, Cairo University (Cairo, Egypt; PT 2613). All efforts were exerted to minimize animal suffering during the experimental period.

### 2.2. Experimental Design

**Figure 1:**
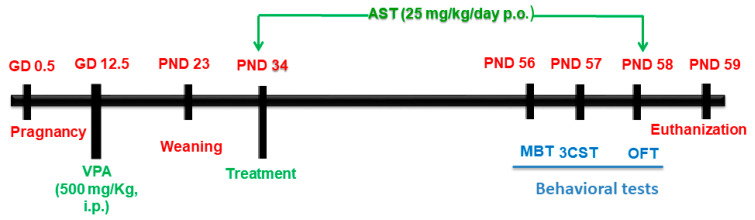
Schematic timeline for prenatal VPA model induction, AST treatment schedule, behavioral experiments, and euthanization. AST: astaxanthin; 3-CST: three-chamber sociability test; GD: gestational day; MBT: marble burying test; OFT: open field test; PND: postnatal day.

On PND 34 (early adolescence), animals were allocated into 3 groups containing 9 rats each. The in utero saline-injected male offspring (group 1) served as the control group (CONT), whereas VPA-exposed male offspring were randomly allocated into two groups (2 and 3). Animals in group 2 were left untreated and designated as the model group (ASD), whereas those in group 3, were administered AST daily (25 mg/kg; Nutrex Hawaii Inc., Kailua-Kona, HI, USA) [63] until PND 58 (early adulthood). Animals in the first two groups (CONT and ASD) received distilled water p.o daily for 25 days (PND 58). Starting from PND 56, behavioral tests were performed. The marble burying test (MBT) was conducted to assess repetitive, stereotyped behavior, whereas the three-chamber test (3CT) was carried out on PND 57 to assess sociability and social novelty preference. Finally, general locomotor activity was assessed in open field apparatus on PND 58 (Figure 1).

### 2.3. Assessment of Autistic-Like Behaviors

#### 2.3.1. Marble-Burying Test (MBT)

In this test, rats were placed individually in a standard Plexiglas cage containing a 5 cm depth of clean bedding. Fifteen glass marbles (15 mm diameter) were evenly placed in 3 rows on the bedding, and animals were tested for 20 min for burying/hiding behavior. The number of marbles buried with bedding up to two-thirds of their depth was counted [64,65].

#### 2.3.2. Three-Chamber Sociability Test

The test was conducted as mentioned before [60,61,66]. The three-chamber apparatus (60 × 45 × 45 cm) is divided into three connected chambers with openings between the compartments, allowing the animals to freely explore the three chambers, and a wire cage is placed in each of the lateral compartments. Notably, the chambers were cleaned with 70% alcohol after each tested animal. The 3-CST consisted of three phases: the habituation phase, in which the tested rat was placed in the central chamber before the sociability phase and was allowed to explore for 5 min. The second is the sociability phase, which started directly after the habituation phase, where a novel rat (novel rat 1) of the same age, sex, and strain was placed into a wire cage in one of the lateral chambers, and the other wire cage was left empty, representing the novel object. The tested rat was left for 10 min, and the time spent exploring novel rat 1 or the novel object was recorded. The sociability index (*SI*) was calculated as follows:

(1) $$SI=\frac{\left(time\;exploring\;novel\;rat\;1-time\;exploring\;novel\;object\right)}{\left(time\;exploring\;novel\;rat\;1+time\;exploring\;novel\;object\right)}$$

The third phase is the social novelty preference phase, which starts directly after the sociability phase for an additional 10 min. A new, unfamiliar rat was placed into the opposite chamber’s empty wire cage and considered “novel rat 2”. This session evaluates the preference for the social novelty of the tested rat to choose whether to interact with the familiar animal (novel rat 1) or the novel one (novel rat 2). The social novelty preference index (*SNI*) was calculated using the following equation:

(2) $$SNI=\frac{\left(time\;exploring\;novel\;rat\;2-time\;exploring\;familiar\;rat\right)}{\left(time\;exploring\;novel\;rat\;2+time\;exploring\;familiar\;rat\right)}$$

#### 2.3.3. Open Field Test (OFT)

A 60 × 60 × 45 cm quadrangular arena divided into 16 equal squares was used. Rats were allowed to acclimatize to the test room for 30 min before starting the experiment, and then each rat was individually placed in the center of the dimly illuminated arena for 5 min. The number of crossed squares visited with all 4 feet, the entries to the central zone compromising the central 4 squares, the time spent in this zone, and the latency to leave it were recorded. In addition, the duration (s) of rearing and grooming was documented. The test arena was cleaned with 70% alcohol after each rat [67,68]. 

### 2.4. Cortical Processing

After behavioral testing, all animals were euthanized under deep anesthesia (Phenobarbital 100 mg/kg) followed by transcardiac perfusion with phosphate-buffered saline (PBS; pH 7.4), and the cortices were rapidly dissected and divided into 3 sets (each containing 3 rats). The left hemispheres of animals in the first set were fixed in 10% neutral buffered formalin for histopathological/immunochemical examinations, whereas those of animals in the second set were kept in glutaraldehyde for electron microscopy. From 3 animals in set 3, the left cortices were submerged in a RIPA buffer with protease and phosphatase inhibitors for measurements with Western blots. After that, the right cortex of the first 3 rats (set 1) was placed in lysis buffer for qRT-PCR analysis, and the remaining right cortices from rats in sets 2 and 3 were flash frozen in liquid nitrogen and then homogenized in PBS and aliquoted for the assessment of ELISA parameters and stored at −80 °C. 

#### 2.4.1. Assessments of *p*^(S396)^-tau, *p*^(Y458/199)^-PI3K p85/p55, Notch1, NICD, p25, and p35 Using the Western Blot Technique

Following cortical protein quantification (Bio-Rad Protein Assay Kit, Hercules, CA, USA), 10 μg protein of each sample was separated using SDS polyacrylamide gel electrophoresis, transferred to a PVDF membrane, and then blocked with 5% bovine serum albumin (BSA). Afterward, the membrane was incubated with anti-*p*^(S396)^-tau (cat#: 35-5300), anti- *p^(^*^Y458/199)^-PI3K p85/p55 (cat#: PA5-17387), anti-Notch1 (cat#: PA5-23181), anti-NICD-1 (cat#: PA5-99448), anti-P35 (cat#: MA5-14834), or β-actin (cat#: PA1-183) polyclonal antibody (ThermoFisher Scientific, Waltham, MA, USA) and anti-P25 (ABClonal Technology, Woburn, MA, USA; cat#: A4637) overnight at 4 °C on a roller shaker. Next, the membranes were probed with horseradish peroxidase-conjugated goat anti-rabbit immunoglobulin (Dianova, Hamburg, Germany). Finally, the blots were developed with an enhanced chemiluminescence detection reagent (Amersham Biosciences, Chicago, IL, USA), and the corresponding expressed protein was quantified with densitometric analysis using a scanning laser densitometer (GS-800 system, Bio-Rad, Hercules, CA, USA). The results were expressed as arbitrary units (AUs) after normalization to β-actin.

#### 2.4.2. Assessments of Cortical Gene Expressions of Mas Receptor with qRT-PCR

Total RNA was extracted from cortical tissue using an RNeasy Mini kit (Qiagen, Venlo, Netherlands), and the obtained RNA was verified for pureness spectrophotometrically at OD 260/280 nm. According to the manufacturer’s procedure, equal amounts of RNA (1 μg) were reverse transcribed into cDNA using a reverse transcription system (Promega, Leiden, Netherlands). The qRT-PCR of the Mas receptor was performed using SYBR Green Master Mix (Applied Biosystems, Waltham, CA, USA). In a 25 μL reaction volume, 5 μL of cDNA was combined with 12.5 μL SYBR Green mixture, 5.5 μL RNase-free water, and 2 μL of each primer (Table 1). The PCR amplifications were accomplished with 40 cycles for 15 s at 95 °C (denaturation), 60 s at 60 °C (annealing), and 60 s at 72 °C (extension). The relative expression of the target gene was obtained using the 2^−ΔΔCT^ formula using β-actin as a housekeeping gene.

#### 2.4.3. Quantification of Cortical Contents of Ang II, ACE2, and Ang1-7 Using the ELISA Technique

MyBioSource ELISA kits (San Diego, CA, USA) were used for the determination of Ang II (cat#: MBS705139), ACE2 (cat#: MBS014209), and Ang1-7 (cat#: MBS2604372). All procedures were performed according to the manufacturer’s instructions.

### 2.5. Microscopic Investigation

For both histopathological and immunohistochemistry inspections, brain specimens were embedded in paraffin, and sections of 5 μm thickness were blindly inspected by a pathologist under a light microscope (BX43, Olympus, Tokyo, Japan) and photographed using Cellsens dimension software version 4.1 CS-EN-V4.1 (Olympus) connected to an Olympus DP27 camera. The Nissl staining and immune reactivity of the corresponding antigens were assessed using image analysis software (Image J, version 1.46a, NIH, MD, USA). Measurements were prepared from five non-overlapping randomly chosen fields in each section at 200× magnification and averaged.

#### 2.5.1. Hematoxylin and Eosin Staining

Brain sections were stained with hematoxylin and eosin (H & E), and the cortical neuropathologic damage was graded from 0 to 4 as follows: (0) indicated no changes; (1) indicated <10% area affected; (2) indicated 20–30% area affected; (3) indicated 40–60% area affected; and (4) indicated >60% area affected [69].

#### 2.5.2. Nissl Staining

Brain sections were stained with cresyl violet to analyze the intensity of the Nissl proteins in cortical neurons. Brain sections mounted on gelatinized slides were air-dried and stained with 0.1% aqueous cresyl violet (Sigma-Aldrich, St. Louis, MO, USA) for 20 min at 60 °C. The slides were then washed in distilled water, differentiated in 70% ethyl alcohol, dehydrated in ascending grades of ethyl alcohol, and cleared in xylene to be mounted with DPX (Sigma-Aldrich). The number of both intact and degenerated neurons was counted. 

#### 2.5.3. Cortical GFAP and NF-κB p65 Immunoreactivity

The deparaffinized and rehydrated sections were incubated with primary rabbit monoclonal GFAP antibody (Cat#: SAB4501162; Merck KGaA, ON, Canada) or primary mouse monoclonal nuclear factor-kappa B (NF-κB) p65 (cat #: sc-8008; Santa Cruz Biotechnology Inc., Dallas, TX, USA) antibody. Subsequently, they were washed with PBS and incubated with a secondary antibody using the HRP EnVision kit (DAKO, Santa Clara, CA, USA). Sections were developed and visualized using diaminobenzidine tetrachloride. The substrate system produced a crisp brown end-product at the site of the target antigen. Sections were counterstained with hematoxylin, dehydrated in alcohol, cleared in xylene, and covered and slipped for microscopic examination. GFAP and NF-κB p65 quantification was estimated by measuring the area% immune expression.

#### 2.5.4. Electron Microscopy

For transmission electron microscopy (TEM) preparation, the samples were fixed in 3% glutaraldehyde in 0.1 M sodium cacodylate buffer (pH = 7.0) for 2 h at room temperature, rinsed in the same buffer, and post-fixed in 1% osmium tetroxide for 2 h at room temperature. The samples were dehydrated in an ethanol series ranging from 10% to 90% for 15 min in each alcohol dilution and finally with absolute ethanol for 30 min. The samples were infiltrated with epoxy resin and acetone through a graded series until they were finally in pure resin. Ultrathin sections were collected on formvar-coated copper grids. The sections were then double stained in uranyl acetate followed by lead citrate. The stained sections were observed with a JEOL JEM 1010 transmission electron microscope at 70 kV at the Regional Center for Mycology and Biotechnology (RCMB), Al-Azhar University (Cairo, Egypt).

### 2.6. Statistical Analysis

For parametric data, values were expressed as mean ± SD, and statistical differences between groups were computed using a one-way analysis of variance (ANOVA) followed by Tukey’s multiple comparisons tests. For non-parametric data, values were expressed as median (minimum-maximum) and analyzed using a Kruskal–Wallis test followed by a Dunnett’s post hoc or Mann–Whitney test to compare every 2 groups. The level of significance was set at *p* < 0.05. GraphPad Prism version 9.4.1 (GraphPad Software Inc., San Diego, CA, USA) software was used to carry out all the statistical tests and to draw the corresponding figures.

## 3. Results

### 3.1. AST Increases Sociability While Reducing Repetitive/Compulsive Behaviors in Autistic Rats

**Figure 2:**
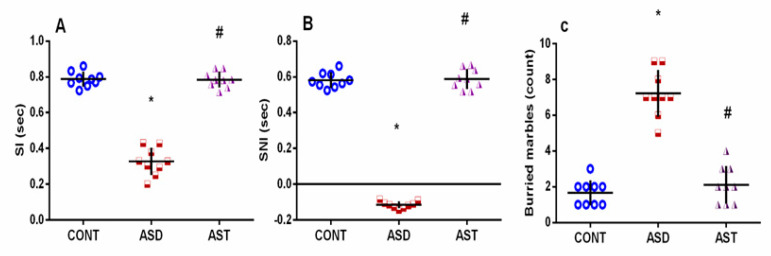
Effect of AST on (**A**) SI and (**B**) SNI in 3-CST, and (**C**) MBT in autistic rats. Male offspring rats of dams were injected with VPA on GD 12.5 and were treated with AST (25 mg/kg/day, p.o) on PND 34 (early adolescence) until PND 58 (early adulthood). Values are expressed as mean ± SD (*n* = 9 rats/group). Statistical analysis was carried out using one-way ANOVA followed by Tukey’s post hoc test, *p* < 0.05, as compared with the CONT (*) and ASD (#) groups. ANOVA: analysis of variance; AST: astaxanthin; ASD: autism spectrum disorder; CONT: control; 3-CST: three-chamber sociability test; GD: gestational day; MBT: marble burying test; p.o: per os; PND: postnatal day; SI: sociability index; SNI: social novelty preference index; VPA: valproic acid. Blue circles for control group; red squares for ASD group; purple triangles for AST treated group are indicative of individual data.

**Figure 3:**
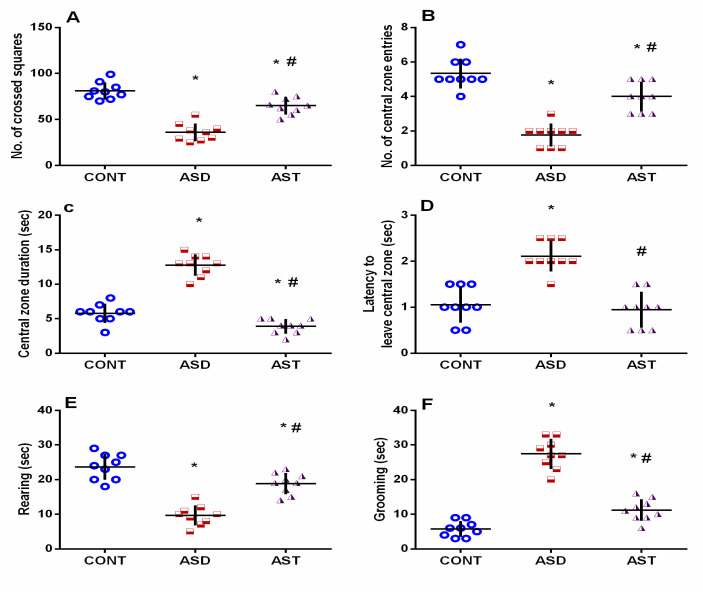
Effect of AST on locomotor behavior and exploration of surroundings using the OFT test in autistic rats: (**A**) the total number of crossed squares; (**B**) frequency of entering the central zone; (**C**) stay duration in the central zone; (**D**) latency to leave the central zone; (**E**) duration of rearing; and (**F**) grooming. Male offspring rats of dams were injected with VPA on GD 12.5 and were treated with AST (25 mg/kg/day, p.o) on PND 34 (early adolescence) until PND 58 (early adulthood). Values are expressed as mean ± SD (*n* = 9 rats/group). Statistical analysis was carried out using one-way ANOVA followed by Tukey’s post hoc test, *p* < 0.05, as compared with the CONT (*) and ASD (#) groups. ANOVA: analysis of variance; AST: astaxanthin; ASD: autism spectrum disorder; CONT: control; GD: gestational day; OFT: open field test; p.o: per os; PND: postnatal day; VPA: valproic acid. Blue circles for control group; red squares for ASD group; purple triangles for AST treated group are indicative of individual data.

Both impaired social and repetitive/compulsive behaviors are diagnostic hallmarks of ASD. During the 3-CST, male autistic rats (Figure 2A) displayed significant reductions in the SI (A) and SNI (B) by 59% (*F*_(2,24)_ = 196.7, *p* < 0.0001) and 120% (*F*_(2,24)_ = 818.5, *p* < 0.0001), respectively compared with the CONT group. However, socialization improvement was noted following AST treatment, where the carotenoid abrogated social withdrawal and normalized both SI (*F*_(2,24)_ = 196.7, *p* < 0.0001) and SNI (*F*_(2,24)_ = 818.5, *p* < 0.0001) in VPA in utero exposed offspring. Additionally, VPA exposure enhanced repetitive stereotyping in the marble burying test, as indicated by the 4-fold (*F*_(2,24)_ = 77.85, *p* < 0.0001) increment in the number of hidden marbles (C) compared with the CONT group. This repetitive compulsive-like disorder was, however, normalized in the AST-treated group, indicating reduced disruptive behavior and social discourse difficulties (*F*_(2,24)_ = 77.85, *p* < 0.0001). A defective exploration of the surroundings was evaluated with the OFT (Figure 3) and manifested by the significant decrease in the total number of crossed squares (A) by 55% (*F*_(2,24)_ = 51.56, *p* < 0.0001), along with the entries to the central zone (B) by 67% (*F*_(2,24)_ = 44.80, *p* < 0.0001). These decrements were associated with a 2-fold (*F*_(2,24)_ = 107.7, *p* < 0.0001) prolongation of time spent in the central zone (C) and latency (D) (2 folds; *F*_(2,24)_ = 26.87, *p* < 0.0001) along with a reduction in rearing behavior (E) by 59% (*F*_(2,24)_ = 42.92, *p* < 0.0001). In addition, animals experienced a longer duration of grooming (F) (4.7 folds; *F*_(2,24)_ = 100.4, *p* < 0.0001), denoting higher repetitive stereotyped behaviors compared with the CONT group. In contrast, AST-enhanced locomotion was indicated by a large number of crossed squares (1.8 folds; *F*_(2,24)_ = 51.56, *p* < 0.0001) and entries to the central zone (2.2 folds; *F*_(2,24)_ = 44.80, *p* < 0.0001) with a shorter time spent in the central zone (30%; *F*_(2,24)_ = 107.7, *p* < 0.0001) compared with the VPA untreated group. Additionally, AST normalized the latency to leave the central zone (45%; *F*_(2,24)_ = 26.87, *p* < 0.0001), increased rearing to 2 folds (*F*_(2,24)_ = 42.92, *p* < 0.0001), and abrogated grooming (41%; *F*_(2,24)_ = 100.4, *p* < 0.0001), as compared with the ASD group. The amendments mentioned above in AST-treated rats signify the ability of AST to improve sociability and reduce repetitive/compulsive core signs of autism.

### 3.2. AST Modulates Cortical AngII/ACE2/Ang1-7/Mas Receptor/Trajectory and Augments PI3K p85/p55 in Autistic Rats

**Figure 4:**
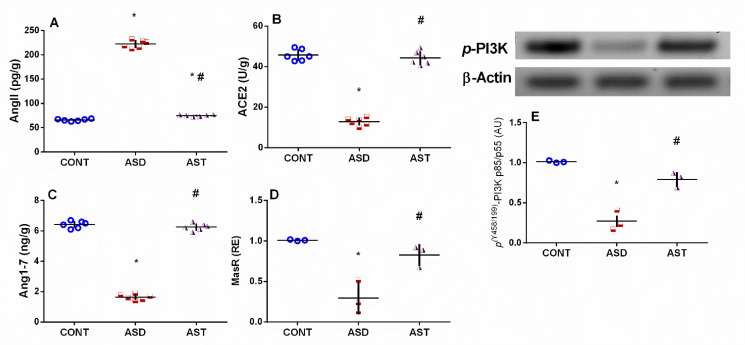
Effect of AST on cortical contents/gene expression of (**A**) Ang II, (**B**) ACE2, (**C**) Ang1-7, (**D**) MasR, and (**E**) p(Y458/199)-PI3K p85/p55 of autistic rats. Male offspring rats of dams were injected with VPA on GD 12.5 and were treated with AST (25 mg/kg/day, p.o) on PND 34 (early adolescence) until PND 58 (early adulthood). Values are expressed as mean ± SD (*n* = 3 rats/group for PCR and 6 rats/group for ELISA). Statistical analysis was carried out using one-way ANOVA followed by Tukey’s post hoc test, *p* < 0.05, as compared with the CONT (*) and ASD (#) groups. ANOVA: analysis of variance; AST: astaxanthin; ASD: autism spectrum disorder; CONT: control; ACE2: angiotensin-converting enzyme 2; Ang1–7: angiotensin 1–7; Ang II: angiotensin 2; MasR: Mas receptor; p.o: per os; p(Y458/199)-PI3K: phosphorylated phosphoinositide 3-kinase at tyrosine 458 and 199; PND: postnatal day; VPA: valproic acid; GD: gestational day. Blue circles for control group; red squares for ASD group; purple triangles for AST treated group are indicative of individual data.

As depicted in Figure 4, the ASD model resulted in a significant increase of 3.4-fold (*F*_(2,15)_ = 1887, *p* < 0.0001) in the cortical content of Ang II (A), a vital activator of the canonical RAS. However, ASD reduced ACE2 (B), an enzyme that catalyzes the hydrolysis of Ang II into Ang1-7 for homeostatic regulation, as well as Ang1-7 (C), a heptapeptide that counterbalances the Ang II harmful effects, by 72% (*F*_(2,15)_ = 264.8, *p* < 0.0001) and 74% (*F*_(2,15)_ = 892.1, *p* < 0.0001), respectively. Additionally, the mRNA expression of Mas receptor (D), the Ang1-7 receptor that activates the alternative protective RAS arm, was downregulated by 71% (*F*_(2,6)_ = 20.87, *p* = 0.0019). Furthermore, the insult led to a depletion of 73% in the protein expression of (E) *p^(Y458/199)^*-PI3K p85/p55 (*F*_(2,6)_ = 53.63, *p* = 0.0001), a downstream transducer for the Mas receptor signaling. However, treatment with AST effectively corrected the altered molecules of RAS and increased PI3K protein expression to nearly normal levels across most parameters. Hence, modulation of the Yin/Yang RAS axis can be a novel player in the pathomechanism of ASD and can be one of the anti-autistic mechanisms of AST by reducing Ang II, activating the ACE2 enzyme to trigger the Ang1-7/Mas receptor axis, and stimulating its neuroprotective downstream target PI3K p85/p55.

### 3.3. AST Deactivates the Cortical Notch1/NICD-1/NF-κB Trajectory and Increases the Cortical p25/p35 Ratio in Autistic Rats

**Figure 5:**
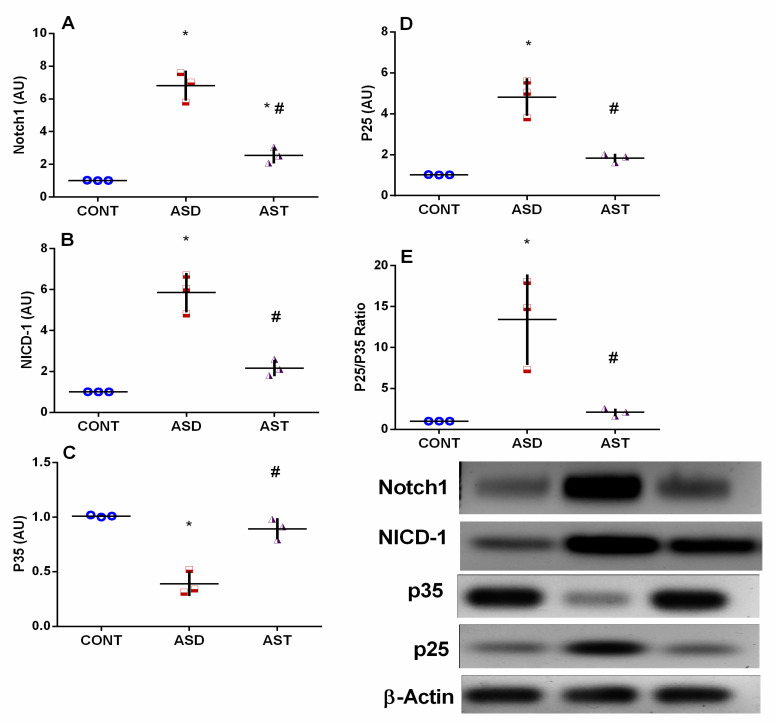
Effect of AST on the protein expression of (**A**) Notch1, (**B**) NICD, (**C**) p35, (**D**) p25, and their ratio (**E**) p25/35 in the cerebral cortex of autistic rats. Male offspring rats of dams were injected with VPA on GD 12.5 and were treated with AST (25 mg/kg/day, p.o) on PND 34 (early adolescence) until PND 58 (early adulthood). Values are expressed as mean ± SD (*n* = 3 rats/group). Statistical analysis was carried out using one-way ANOVA followed by Tukey’s post hoc test, *p* < 0.05, as compared with the CONT (*) and ASD (#) groups. ANOVA: analysis of variance; AST: astaxanthin; ASD: autism spectrum disorder; CONT: control; GD: gestational day; Notch1: neurogenic locus notch homolog protein 1; NICD: Notch intracellular domain; p.o: per os; PND: postnatal day; VPA: valproic acid. Blue circles for control group; red squares for ASD group; purple triangles for AST treated group are indicative of individual data.

Figure 5 shows that the autistic rats displayed an elevated protein expression of Notch1 (A), a single-pass transmembrane receptor activated by Ang II and CDK5/p25 kinase, and its downstream intracellular signaling molecule NICD-1 (B). These parameters were increased by approximately 6.7 fold (F_(2,6)_ = 74.80, *p* < 0.0001) and 6 fold (F_(2,6)_ = 52.58, *p* = 0.0002), respectively. Additionally, the insult led to a downregulation in the protein expression of the neuron-specific CDK5 activator (C), p35, by 61%, while upregulating p25 (D) generated by Ca^2+^-dependent calpain cleavage of p35, to approximately 4.7 fold, resulting in a significant increase in the p25/p35 ratio (E) by 13.4-fold. However, treatment with AST effectively inhibited the protein expression of the Notch1/NICD-1 axis and reversed the impact of VPA on the p25/p35 ratio by increasing p35 (2.3-fold) while reducing p25 and the p25/p35 ratio by 62% and 84%, respectively, compared with the insulted group. 

**Figure 6:**
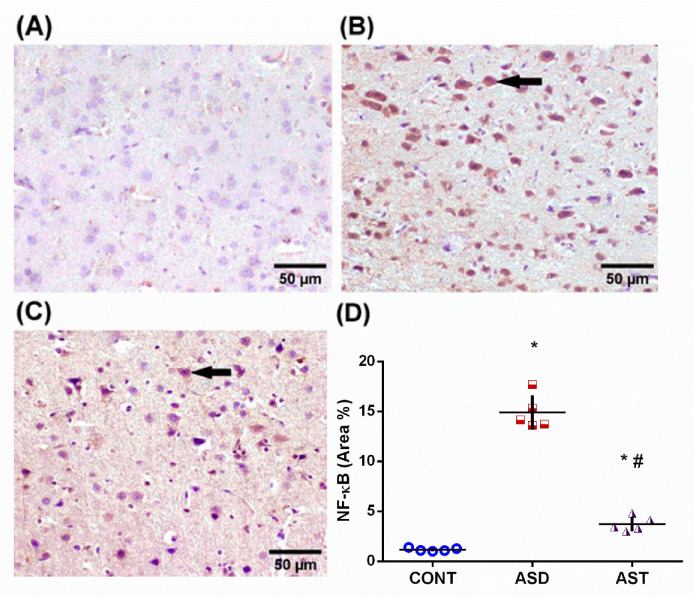
Representative photomicrographs of NF-κB immuno-expression in the cerebral cortex illustrating the differences between the groups. Section of the CONT (**A**) group shows negative immune expression, while that from the ASD (**B**) rats depicts strong immunoreactivity (arrow). Section from the AST-treated group (**C**) represents a weak NF-κB immune reaction (arrow; scale bar 50 μm). The data in panel (**D**) represent the mean ± SD, with 5 fields analyzed from 3 rats per group. Statistical analysis was conducted using one-way ANOVA followed by Tukey’s post hoc test, with *p* < 0.05 considered significant. (*) and (#) indicate comparisons with the CONT and ASD groups, respectively. ANOVA: analysis of variance; AST: astaxanthin; ASD: autism spectrum disorder; CONT: control; NF-κB: nuclear factor kappa B. Blue circles for control group; red squares for ASD group; purple triangles for AST treated group are indicative of individual data.

ASD-induced activation of the Notch1/NICD-1 axis extended to impact its downstream target NF-κB, a transcription factor with pleiotropic effects, as presented in Figure 6. The ASD insult resulted in an increased immunoreactivity of the transcription factor NF-κB, reaching a level approximately 12.7-fold higher (*F*_(2,12)_ = 232.0, *p* < 0.0001) compared with the CONT group (A, D), whereas treatment with AST (C, D) blunted this expression by 75% (*F*_(2,12)_ = 232.0, *p* < 0.0001). These results confirmed the involvement of the active inflammatory signal Notch1/NICD-1/NF-κB in the ASD model and the inhibited CDK5, which plays a role in synaptic plasticity and the formation of learning and memory. Oppositely, AST acted by reverting the ASD effect to enhance the neurogenic CDK5 indicated by stimulation of p35 in addition to abating the inflammatory trajectory.

### 3.4. AST Reduces ASD Hallmarks Astrogliosis/p-tau, Amends Cortical Structure, and Rescues Neurons in Autistic Rats

**Figure 7:**
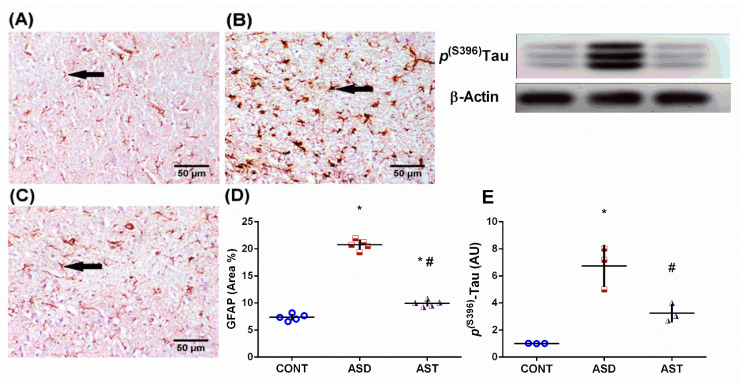
Effect of AST on GFAP and p(ser396)-tau protein expression. Representative photomicrographs of cortical GFAP immuno-expression in the CONT (**A**) rats showing normal small-sized astrocytes with lightly stained GFAP positive-short processes. In contrast, the section from the ASD rats (**B**) displays astrogliosis, indicated by strongly immunoreactive hypertrophied astrocytes with deeply stained GFAP-positive brown processes. However, the section from the AST-treated rats (**C**) demonstrates a soothing effect on this reaction, with mild GFAP-positive expression (scale bar = 50 μm). The data are summarized in panel (**D**). Panel (**E**) presents the protein expression of p(ser396)-tau. The data are presented as mean ± SD, with 5 fields analyzed from 3 rats per group for GFAP and 3 rats per group for tau. Statistical analysis was performed using one-way ANOVA followed by Tukey’s post hoc test, with *p* < 0.05 considered significant. (*) and (#) indicate comparisons with the CONT and ASD groups, respectively ANOVA: analysis of variance; AST: astaxanthin; ASD: autism spectrum disorder; CONT: control; GFAP: glial fibrillary acidic protein; p(ser396)-tau: phosphorylated tau protein at serine 396; p.o: per os; PND: postnatal day; VPA: valproic acid. Blue circles for control group; red squares for ASD group; purple triangles for AST treated group are indicative of individual data.

Figure 7 shows strong immunoreactivity of the astrocyte marker GFAP in the section of (B) autistic rats that shows deep brown-stained hypertrophied astrocytes in the cerebral cortex compared with the lightly stained section of the CONT rats (A) that have normal small-sized astrocytes. Moreover, AST (C) treatment weakened astrogliosis, as evidenced by the light GFAP immunoreactivity, compared with the ASD section. The data are summarized in panel D (*F*_(2,12)_ = 453.8, *p* < 0.0001). Moreover, the ASD insult caused a 6.6-fold increase in the protein expression of p^(S396)^-tau (E), which disables neural network function in ASD to be significantly downregulated upon treatment with AST.

**Figure 8:**
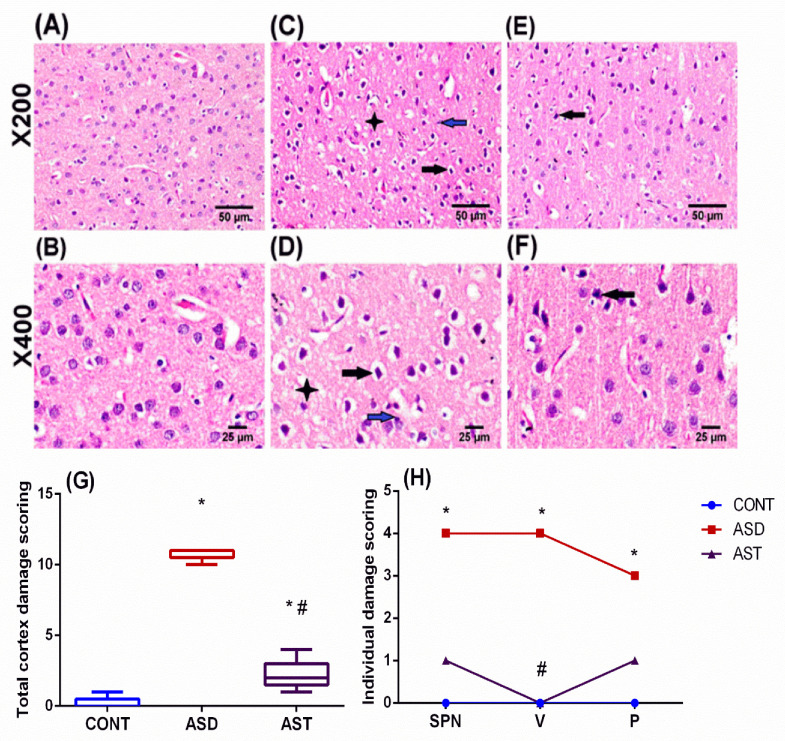
Representative photomicrographs of H & E-stained cerebral cortexes of rats in the CONT group (**A**,**B**) show normal histological architecture with intact neurons, whereas sections of the ASD rats (**C**,**D**) show pyknosis with shrunken and reduced neuronal size (black arrow), vacuolation of neuropil (asterisk), and proliferation of glial cells (blue arrow). Sections from the AST-treated rats (**E**,**F**) depict a few shrunken necrotic neurons (black arrow) with a mild proliferation of glial cells (scale bar, 50 and 25μm). Panel (**G**) illustrates the collective cortical damage scores presented as box and whiskers; median (minimum–maximum) data were analyzed using the Mann–Whitney test to compare between every 2 groups. Panel (**H**) depicts individual cortical alteration scores expressed as median (max–min) and analyzed using the Kruskal–Wallis test followed by Dunnett’s posthoc test. Values were calculated from 5 fields of 3 rats/group (*p* < 0.05), as compared with the CONT (*) and ASD (#) groups. Male offspring rats of dams were injected with VPA on GD 12.5 and were treated with AST (25 mg/kg/day, p.o) on PND 34 (early adolescence) until PND 58 (early adulthood). AST: astaxanthin; ASD: autism spectrum disorder; CONT: control; GD: gestational day; P: proliferation of glial cells; p.o: per os; PND: postnatal; SPN: shrunken, pyknosis, and necrosis of neurons; V: vacuolation of neuropil; VPA: valproic acid; Blue circles for control group; red squares for ASD group; purple triangles for AST treated group are indicative of individual data.

The photomicrographs (Figure 8) of ASD rats (C, D) showed severe neuropathic lesions surrounded by empty areas and were characterized by increased cell-packing density, pyknosis, and reduced neuronal size. In addition, vacuolation of neuropil and proliferation of glial cells were noted compared with CONT rats (A, B), which exhibited normal histological architecture with intact neurons. On the contrary, sections of the AST-treated rats (E, F) depicted a marked upturn with some shrunken necrotic neurons associated with mild proliferation of glial cells. As shown in panel G, ASD scores reached 11 (*p* = 0.0010), whereas AST abated it to 2 (*p* = 0.0079); furthermore, it improved the individual lesions, as presented in panel H.

**Figure 9:**
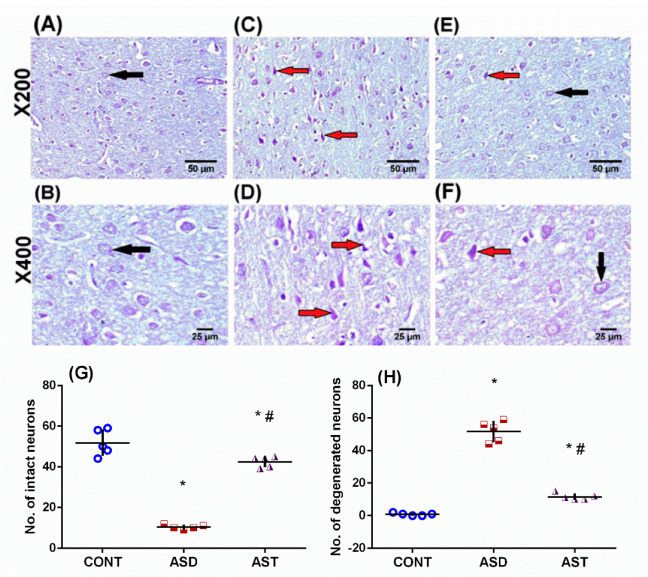
Representative photomicrographs of cresyl violet-stained sections of the cerebral cortex illustrate the differences between the groups. In the CONT group (**A**,**B**), normal intact neurons with visible nuclei are observed, and Nissl proteins around the nucleus are stained blue-violet (black arrow). Sections from the ASD rats (**C**,**D**) show shrunken, darkly stained neurons (red arrow), while sections from the AST-treated group (**E**,**F**) display a decreased number of degenerated, darkly stained neurons (red arrow) and an increased number of intact neurons (black arrow) (scale bar, 50 and 25 μm). The panels (**G**,**H**) provide quantitative data on the number of intact and degenerated neurons, respectively. The values are expressed as mean ± SD, with 5 fields of view analyzed from 3 rats per group. Statistical analysis was conducted using one-way ANOVA followed by Tukey’s multiple comparisons tests, with *p* < 0.05 considered significant. (*) and (#) indicate comparisons with the CONT and ASD groups, respectively. Male offspring rats of dams were injected with VPA on GD 12.5 and were treated with AST (25 mg/kg/day, p.o) from PND 34 (early adolescence) until PND 58 (early adulthood). ANOVA: analysis of variance; ASD: autism spectrum disorder; AST: astaxanthin; CONT: control, GD: gestational day; p.o: to per os; PND: postnatal day; VPA: valproic acid. Blue circles for control group; red squares for ASD group; purple triangles for AST treated group are indicative of individual data.

Sections stained with Nissl stain (Figure 9) revealed that ASD rats (C, D) possess shrunken and darkly stained neurons, as compared with sections of the CONT rats (A, B) that depict intact neurons with a visible nucleus and blue-violet stained perinuclear Nissl proteins. Furthermore, the AST-treated rat (E, F) sections revealed decreased degenerated neuronal cells. Panels G (*F*_(2,12)_ = 139.1, *p* < 0.0001) and H (*F*_(2,12)_ = 229.5, *p* < 0.0001) represent the number of intact and degenerated neurons, respectively.

**Figure 10:**
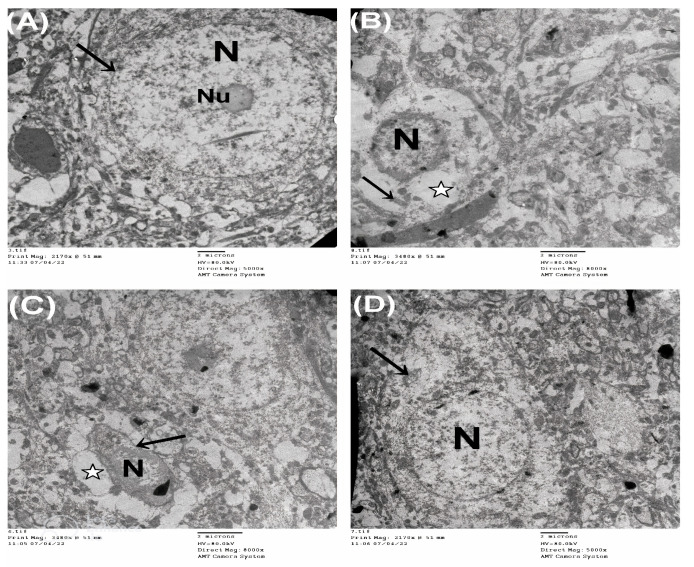
Electron micrographs of cerebral cortex sections from different groups provide insights into cellular changes. In the CONT group (**A**), the images show normal nuclei (N) with regular nuclear membranes (arrow) and prominent nucleoli (Nu). In contrast, the ASD group (**B**) exhibits a shrunken nucleus (N), empty lysed cytoplasm (white star), and degenerated mitochondria (arrow). Similarly, section (**C**) displays an uneven nucleus (N) surrounded by an irregular nuclear membrane (arrow) and lysed cytoplasm (white star). However, in the AST-treated group (**D**), the section reveals a large, normal nucleus (N) surrounded by apparently normal cytoplasm containing slightly swollen mitochondria (arrow).

In Figure 10, electron micrographs of cortical sections of CONT rats (A) show normal nuclei with regular nuclear membranes and prominent nucleoli. In contrast, the sections of the ASD model (B, C) depict a shrunken irregular nucleus with an irregular nuclear membrane, empty lysed cytoplasm, and degenerated mitochondria. However, the section of the AST-treated rats (D) shows a large normal nucleus surrounded by apparently normal cytoplasm containing slightly swollen mitochondria. Accordingly, the enhanced cortical structure and neuronal count by AST is a consequence of its ability to deter astrogliosis and p-tau, two decisive pathological events related to autism.

To sum up, AST, by activating the Mas receptor and stimulating ACE2-induced Ang1-7 production, halted Ang II, Notch1/NICD1/p25, and NF-κB hubs. Additionally, AST enhanced two survival molecules, namely, PI3K and p35. The crosstalk between these proteins amended structural alterations and behavioral deficits induced by the VPA-induced autistic model. Moreover, the interplay between these signals limits both tauopathy and astrogliosis, which participate in the pathomechanism of ASD.

## 4. Discussion

ASD is a complex neurodevelopmental disorder characterized by difficulties in social interaction, communication, and repetitive behaviors. Despite its prevalence, the pathophysiology of ASD is not well understood, making it challenging to develop effective treatments. The underlying causes of ASD are believed to be a combination of genetic and environmental factors, but specific mechanisms are still unclear. This lack of understanding hampers the progress in finding targeted therapies for individuals with ASD, leaving many struggling to find suitable treatments and interventions. As research continues to uncover the underlying mechanisms and causes of ASD, it is hoped that more effective treatments will be developed to improve the lives of those affected by this disorder.

**Figure 11:**
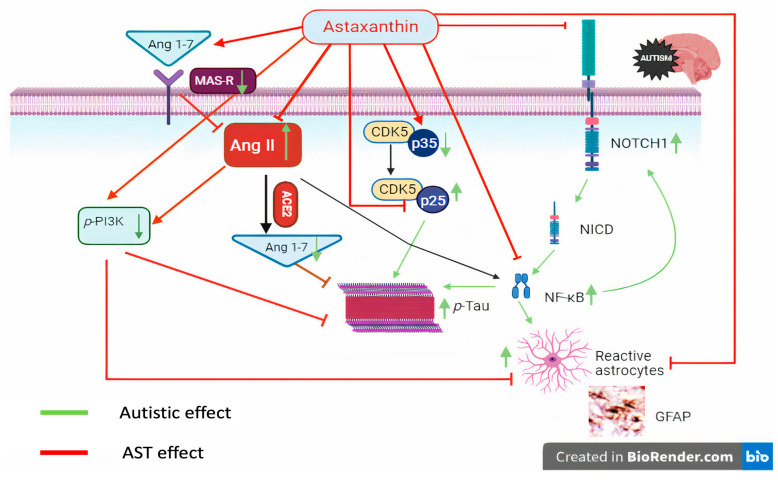
Illustrating diagram representing the effect of AST through interaction with different signaling proteins to mitigate ASD. AST increased Ang 1-7 by binding to its receptor Mas, inhibited Ang II, and activated ACE2 to increase the formation of Ang 1-7 to abate p-tau. In addition, AST and activated Ang 1-7/Mas receptor activated the survival proteins PI3K and p35 to decrease p-tau and GFAP. AST inhibited the inflammatory cascade Notch1/NICD-1/NF-κB to lessen GFAP and p-tau. Ang: angiotensin; ACE2: angiotensin-converting enzyme 2; GFAP: glial fibrillary acidic protein; Notch 1: neurogenic locus notch homolog protein 1; NICD-1: Notch intracellular domain; NF-κB: nuclear factor kappa B p65; PI3K: phosphoinositide 3-kinase; p-tau: phosphorylated tau protein. The green arrows are indication of autistic effect while red inhibitory lines are indication of AST effect.

Our study revealed, for the first time, the involvement of the imbalanced RAS in the pathomechanisms of ASD, and the correction of this imbalance with AST serves as an in vivo “proof of concept”. By redirecting the RAS toward the beneficial arm, as evidenced by the strengthening of the ACE2/Ang1-7/Mas receptor axis, AST effectively diminished the unwanted effects of Ang II and the Notch1/NICD/NF-κB inflammatory cascade. Simultaneously, AST increased the expression of the neurogenesis marker PI3K and improved the p35/p25 ratio. The interplay between these pathways reduced astrogliosis and p-tau, which play a critical role in ASD pathogenesis. Furthermore, on a behavioral level, these events led to an improvement in sociability/social preference and a decrease in stereotypies/perseverative behavior, which are cardinal features of ASD. Additionally, AST facilitated the repair of the histological structure and enhanced neuronal survival, as indicated with H & E stain, Nissel stain, and electron microscopy (Figure 11).

While previous research has linked genetic variations in ACE to autism [37], this study is the first to examine the role of the Yin/Yang arms of RAS in in utero VPA-exposed animals. The results showed that ASD is associated with a surge in Ang II, the surrogate marker of the classical arm of RAS, and a significant suppression of the good RAS arm ACE2/Ang1-7/MasR. It is worth noting that although there are no existing data on the effect of AST on central cortical RAS, our findings on the inhibitory effect of AST on Ang II can be explained by the work of Gao et al. [70]. They reported that chronic infusion of AST in the hypothalamic paraventricular nucleus in spontaneously hypertensive rats reduced the expression of ACE and AT1R and pointed to the ability of AST to hinder both the synthesis of Ang II and its effect.

Moreover, the effect of AST on the neuroprotective arm of the RAS was investigated to provide a comprehensive understanding. The results revealed that the Yang arm of the RAS was significantly activated by treatment with AST to provide one possible explanation for its neuroprotection in ASD pups. Interestingly, the central role of AST on the beneficial axis of the RAS aligns with its central effect observed in spontaneously hypertensive rats [70], where in addition to inhibiting Ang II, the chronic infusion of AST enhanced the expression of ACE2 and the Mas receptor, reinforcing our findings. The modulatory effect of AST on the RAS may partly contribute to the improved behavior observed in our study, as the disturbed Yang arm of the RAS has previously been implicated in impaired cognition [71]. ACE2 knockout rodents have shown impaired cognitive functions, while upregulation of the Mas receptor has been found to improve social interaction and emotions [72]. These findings highlight the ACE2/Ang1-7/Mas receptor pathway as a potential target for managing disturbed behavior in ASD.

The corrective effect of AST on the beneficial aspect of the RAS can be partially attributed to the activation of PI3K, as observed in our study. This is noteworthy because the neuroprotective role of the PI3K/Akt pathway relies, in part, on the inactivation of Ang II, as demonstrated in a model of hypertension-induced cardiac hypertrophy [73] and the stimulation of the Ang1-7/Mas receptor axis. The latter has been reported to activate PI3K [74], which subsequently counteracts the harmful effects of the Ang II/AT1R axis [75]. Indeed, a previous study strengthened our finding since weakening this protein and its downstream trail, Akt-Bcl-xL, enhanced cell death [18]. Furthermore, in a VPA-induced autistic rat model, Nicolini and coauthors [76] found that the PI3K/AKT trajectory was downregulated. Similarly, in BTBR mice, Luo et al. [77] found that the PI3K/Akt axis was inhibited but reactivated when moesin was overexpressed to mitigate autism-associated behaviors.

In addition to rebalancing the RAS, AST treatment has been shown to curb the inflammatory Notch1/NICD-1/NF-κB cascade, shedding light on another neuroprotective mechanism against the ASD model. It is noteworthy that the dysfunctional Notch pathway has a potential correlation with autism. Consistent with our findings, it has been reported that VPA-induced ASD activates the Notch1 signaling, and its inhibition alleviates autistic-like behavior [78]. In addition, activated Notch1 cascade stimulates its downstream target NF-κB, which has been linked to autism, as documented from post-mortem tissue analysis of autistic patients showing increased NF-κB expression [79]. An interplay between Notch1 and NF-κB has been recognized since NF-κB, in addition to being activated by Notch1, enhances the expression of Jagged-1 to activate the Notch signaling in recipient cells [80]. Our findings demonstrated that AST inhibits the Notch1/NICD-1/NF-κB pathway, explaining the increased survival of neurons in the ASD model.

Moreover, the inhibition of NF-κB has been reported to alleviate synaptic deficits associated with ASD [81]. A previous study explained the inhibitory effect of AST on NICD-1 by enhancing the protein numb homolog (Numb), which degrades NICD-1 proteolytically [82]. Additionally, the ability of AST to downregulate NF-κB protein expression aligns with its effects in a model of subarachnoid hemorrhage [83], supporting its potential as an anti-autistic agent by reducing one of the inflammatory markers associated with autism [79].

The current study highlighted the cross-communication and integration between the RAS and the Notch signaling pathways. In a previous report, Ang II, which is activated by ACE [84], was reported to stimulate both the Notch system [45] and its downstream target NF-κB [85]. Mutually, increased levels of Ang II have been shown to cause Notch1 cleavage and subsequent release of NICD-1 in podocytes [45]. Thus, the reduction in the cortical Ang II by AST is considered a novel culprit for deactivating the Notch1/NICD-1/NF-κB axis, which together intermingles to mitigate autism.

Since p-tau is a characteristic trait of ASD and its reduction mitigates autism key features [86], the AST modulatory effect on the previous markers explains the reduced p-tau. One contributing factor to the observed decrease in tau levels is the AST-mediated inhibition of Ang II since activation of its synthesizing enzyme ACE is associated with an increase in p-tau, an effect that can be reversed by inhibiting the converting enzyme [32]. Additionally, the aptitude of AST to augment the p35/p25 ratio, as observed in our study, partakes in the activation of CDK5 to promote neurogenesis [87]. Similarly, the activation of PI3K plays a role in tau inhibition by inactivating GSK3β [19,26], which is another reason for the inhibited p-tau. Notably, inflammatory Notch1 signaling was reported to increase p-tau in an AD model, and turning off this cascade was shown to reduce this marker [88]. Therefore, the ability of AST to modulate these mechanisms offers a potential explanation for the inhibited p-tau levels observed in our study.

Increased astrogliosis associated with an increase in GFAP is another participant in ASD [89] that is partly linked to the activated inflammatory Notch1/NICD-1/NF-κB axis. Notably, the dysfunctional Notch pathway has a potential correlation with autism [78], and via its pleiotropic actions, the activated Notch/NICD axis has been reported to promote the formation of GFAP [41] by activating NF-κB p65 [90]. Therefore, AST extends the anti-autistic mechanisms via reducing astrogliosis by repressing Notch1 signaling and NF-κB, as presented in our findings. Moreover, the inhibitory effect of AST on NF-κB was recounted earlier in a model of subarachnoid hemorrhage [91], supporting our results. The modulation of RAS may play a role in the reduced GFAP immunoreactivity, as documented earlier, where blocking AT1R with losartan [92] or inhibiting ACE with perindopril [93] in models of AD have inhibited this astrogliosis marker.

In conclusion, our study unveiled novel molecular mechanisms through which AST can partially improve the core behavioral abnormalities associated with ASD. Our results demonstrated that AST effectively mitigates tauopathy by activating the neuroprotective ACE2/Ang1-7/Mas receptor pathway and the neuroprotective proteins PI3K p85/p55 and p35. Moreover, AST, by inhibiting AngII and the Notch1/NICD/NF-ĸB pathway, leads to reduced levels of p-tau and astrogliosis, as evidenced by decreased GFAP expression. Therefore, by modulating these intersecting pathways, AST plays a crucial role in ameliorating behavioral disabilities and improving the compromised cortical structure observed in VPA-induced ASD. Consequently, our findings suggest that targeting both arms of the RAS, specifically the Ang1-7/Mas receptor and AngII, as well as the inflammatory Notch1/NICD/NF-ĸB signaling pathway, could serve as potential therapeutic approaches for treating ASD.

It is important to acknowledge the challenges and limitations of the present study. While this study highlighted the role of the two arms of RAS in the pathophysiology of ASD, it did not encompass the contribution of other angiotensin metabolites to ASD. Therefore, further validations may be needed to establish the role of the examined angiotensin arms and metabolites in the pathophysiology of ASD and its associated abnormal behaviors. However, these limitations should not overshadow the merits of this study. It is pioneering in investigating the involvement of dysregulated RAS in the pathological environment of ASD.

Furthermore, it sheds light on the interaction between the RAS and Notch signaling pathways, providing a deeper understanding of the complex mechanisms underlying ASD. For future clinical applications and drug development, the findings emphasize the need to consider RAS modulators as potential treatments for ASD. Additionally, the effectiveness of anti-inflammatory drugs that inhibit the inflammatory Notch1/NICD-1/NF-ĸB signaling pathway suggests they could be promising therapeutic approaches for ASD treatment.

## 5. Conclusions

Preclinical studies are essential to gain insights into the disease mechanism and to test novel interventions. Despite its cardiovascular role, the RAS attracted attention as a target in neuronal disorders. Recent reviews highlighted the RAS’s crucial role in AD by hindering Aβ plaque and p-tau accumulation, improving symptoms, and slowing disease progression. The promising preclinical results extended to clinical trials, where blocking AT1R reduced stroke risk, improved cognition, and offered neuroprotection in AD patients.

Considering the similarities between AD and ASD, we evaluated the role of RAS in an ASD VPA acid model and studied the possible role of AST in correcting imbalanced arms of the RAS. Our results uncovered a new pathomechanism of ASD and a neuroprotective role for AST to improve behavior. AST was found to mitigate tauopathy and astrogliosis by activating the neuroprotective arm ACE2/Ang1-7/Mas receptor pathway, as well as the neuroprotective proteins PI3K p85/p55 and p35 while inhibiting AngII and the Notch1/NICD/NF-κB pathway. Although our preclinical study supported our hypothesis, additional experiments involving the modulation of several RAS molecules and their targeted administration to the brain are necessary in the future to validate this strategy. Furthermore, clinical trials are still required to substantiate the approach.

## Figures and Tables

**Table 1:** Primer sequences used for qPCR.

Gene Name	Primer Sequence (5′–3′)	Accession Number
MAS receptor	F: TGACCATTGAACAGATTGCCA R: TGTAGTTTGTGACGGCTGGTG	NM_153722
β-Actin	F: CCCATCTATGAGGGTTACGC R: TTTAATGTCACGCACGATTTC	NM_031144.3

## Data Availability

The datasets generated or analyzed during the current study are available from the corresponding author upon reasonable request.

## Abbreviations

3-CT	Three chamber test
ACE2	Angiotensin-converting enzyme 2
AD	Alzheimer disease
Akt/PKB	Protein kinase B
Ang1-7	Angiotensin 1–7
Ang II	Angiotensin 2
ASD	Autism spectrum disorder
AST	Astaxanthin
CDK5	Cyclin-dependent kinase 5
GD	Gestational day
GFAP	Glial fibrillary acidic protein
MasR	Mas receptor
MBT	Marble burying test
NF-κB p65	Nuclear factor Kappa B p65
NICD	Notch intracellular domain
Notch1	Neurogenic locus notch homolog protein 1
OFT	Open field test
PI3K p85/p55	Phosphoinositide 3-kinase p85/p55
PND	Postnatal day
*p*-Tau	Phosphorylated tau protein
RAS	Renin angiotensin system
SI	Sociability index
SNI	Social novelty preference index
VPA	Valproic acid
